# Search for MicroRNAs Expressed by Intracellular Bacterial Pathogens in Infected Mammalian Cells

**DOI:** 10.1371/journal.pone.0106434

**Published:** 2014-09-03

**Authors:** Yuki Furuse, Ryan Finethy, Hector A. Saka, Ana M. Xet-Mull, Dana M. Sisk, Kristen L. Jurcic Smith, Sunhee Lee, Jörn Coers, Raphael H. Valdivia, David M. Tobin, Bryan R. Cullen

**Affiliations:** Department of Molecular Genetics & Microbiology, Duke University Medical Center, Durham, North Carolina, United States of America; University of Würzburg, Germany

## Abstract

MicroRNAs are expressed by all multicellular organisms and play a critical role as post-transcriptional regulators of gene expression. Moreover, different microRNA species are known to influence the progression of a range of different diseases, including cancer and microbial infections. A number of different human viruses also encode microRNAs that can attenuate cellular innate immune responses and promote viral replication, and a fungal pathogen that infects plants has recently been shown to express microRNAs in infected cells that repress host cell immune responses and promote fungal pathogenesis. Here, we have used deep sequencing of total expressed small RNAs, as well as small RNAs associated with the cellular RNA-induced silencing complex RISC, to search for microRNAs that are potentially expressed by intracellular bacterial pathogens and translocated into infected animal cells. In the case of *Legionella* and *Chlamydia* and the two mycobacterial species *M. smegmatis* and *M. tuberculosis*, we failed to detect any bacterial small RNAs that had the characteristics expected for authentic microRNAs, although large numbers of small RNAs of bacterial origin could be recovered. However, a third mycobacterial species, *M. marinum*, did express an ∼23-nt small RNA that was bound by RISC and derived from an RNA stem-loop with the characteristics expected for a pre-microRNA. While intracellular expression of this candidate bacterial microRNA was too low to effectively repress target mRNA species in infected cultured cells *in vitro*, artificial overexpression of this potential bacterial pre-microRNA did result in the efficient repression of a target mRNA. This bacterial small RNA therefore represents the first candidate microRNA of bacterial origin.

## Introduction

RNA interference (RNAi), first reported in *Caenorhabditis elegans* in 1998 [1], involves the sequential cleavage of long double stranded RNAs (dsRNAs), often of exogenous or even viral origin, by the RNase III enzyme Dicer into ∼22-bp small interfering RNA (siRNA) duplexes, bearing two 2-nt 3′ overhangs, one strand of which is then loaded into the RNA-induced silencing complex RISC [2]–[5]. The siRNA then guides RISC to complementary mRNAs, resulting in their inhibition. MicroRNAs (miRNAs) are similar in structure and function to siRNAs, at least in mammals, but are distinct in that they are encoded within the genome of the cell [6]. Mammalian cells encode >1,000 distinct miRNAs that are initially transcribed as part of one arm of an ∼33-bp RNA stem-loop located in a long, capped, polyadenylated transcript referred to as a primary miRNA (pri-miRNA) [7]. In animal cells, this stem-loop is bound by the nuclear RNase III enzyme Drosha and cleaved ∼22 bp away from the loop to generate an ∼60-nt RNA hairpin, bearing a ∼2-nt 3′ overhang, referred to as the precursor miRNA (pre-miRNA) intermediate. The pre-miRNA is exported to the cytoplasm where it is bound by Dicer, which cleaves ∼22 bp from the base of the pre-miRNA, leaving a second 2-nt 3′ overhang, to generate a short RNA duplex that is analogous in structure to an siRNA duplex [7]. As in the case of siRNAs, one strand of this duplex is incorporated into RISC and guides RISC to complementary mRNA target sites, resulting in the post-transcriptional repression of mRNA function [6]. Target recognition is primarily mediated by 5′ nucleotides 2 through 8 of the miRNA, the so-called seed region, and the 5′ end of a given miRNA is therefore usually highly discrete. In many cases, the other strand of the miRNA duplex, referred to as the passenger or star strand, can also be detected and can be annealed to the miRNA leaving the predicted 2-nt 3′ overhangs. The implied existence of such a duplex intermediate provides strong support for the idea that a recovered 22-nt RNA is indeed a miRNA.

Several viruses have been shown to express high levels of virally encoded miRNAs in infected cells that are believed to facilitate viral replication, at least in part, by repressing host innate antiviral immune responses [8], [9]. Moreover, it was recently reported that small RNAs expressed by a plant fungus regulate cellular gene expression using the host cell's own RNAi machinery and thereby contribute to the pathogenicity of this fungus [10]. In contrast, prokaryotes are not believed to express miRNAs, although they do express a wide array of small, non-coding RNAs (sRNAs) that regulate a diverse set of physiological processes inside the bacterial cell [11], [12]. For example, bacterial sRNAs form ribonucleoproteins that control cellular functions ranging from protein secretion to the recognition of foreign nucleic acids [13]–[15]. Additionally, sRNAs can form sRNA/mRNA duplexes that rapidly alter mRNA stability and translation efficiency and thus allow the microbe to swiftly adapt gene expression in response to environmental stresses such as nutrient starvation, changes in pH or temperature [12]. The ability of bacteria to rapidly adapt to such environmental stresses is of particular importance for the survival of pathogens combating the immune system. Accordingly, bacterial sRNAs are now recognized as critical regulators of bacterial virulence [16], [17]. In contrast to our extensive knowledge about sRNAs operating inside the bacterial cell, the question of whether bacterial pathogens also release sRNAs such as miRNAs to target host molecules has not, to our knowledge, been previously addressed. We therefore set out to explore if intracellular bacterial pathogens might have acquired the ability to express and translocate miRNAs, or miRNA precursor molecules, into the cytoplasm of infected cells in order to subvert the host cell RNAi machinery and promote bacterial replication and pathogenesis. In particular, as intracellular bacteria are located in the cytoplasm, we hypothesized that they might transcribe and then secrete small RNA hairpins, functionally analogous to pre-miRNAs, that might then be subjected to Dicer cleavage and incorporation into RISC. If this was indeed the case, then we would expect to recover high levels of such bacterial miRNAs, and possibly also the cognate passenger strand, by deep sequencing. In the present study, we analyzed the small RNA expression profile in cells infected by several distinct varieties of intracellular bacteria (*Chlamydia*, *Legionella* and *Mycobacteria*) and asked if these cells expressed small RNAs of bacterial origin that are loaded into the host RISC and are able to regulate mRNA expression.

## Materials and Methods

### Cell lines

HeLa CCL-2 cells were cultured in Dulbecco's modified Eagle medium (DMEM) supplemented with 10% fetal bovine serum (FBS) and antibiotics. The murine macrophage cell line RAW264.7 was cultured in RPMI-1640 supplemented with 10% FBS. THP-1 cells were cultured in RPMI-1640 supplemented with 0.25% glucose, 1% HEPES, 1% sodium pyruvate, 10% FBS and 0.05 mM β-mercaptoethanol. THP-1 cells were collected by centrifugation, resuspended, counted and adjusted to 2×10^6^ cells/ml. Then they were treated with PMA and cultured for 24 h prior to bacterial infection.

### *Chlamydia trachomatis* infection

For infections with *Chlamydia trachomatis* strain LGV-L2 434/Bu, we used HeLa CCL2 cells. Infections were carried out in six 150-mm diameter tissue culture dishes by adding a suspension of *C. trachomatis* LGV-L2 434/Bu elementary bodies (EBs) at an MOI of 20. Prior to infection, confluent HeLa monolayers were washed once with PBS, then a suspension of EBs with the appropriate number of infectious units was added to the cells in 5 ml of culture medium and rocked gently for 30 min at room temperature. Cells were then washed once with PBS, replenished with 30 ml of fresh culture medium and returned to the tissue culture incubator. At 42 h post infection, cells were inspected under a microscope (Olympus CK Inverted Tissue Culture Microscope) to confirm that all cells were infected. Just before preparation of lysates for immunoprecipitation, or TRIzol lysis and small RNA sequencing, cells were washed twice with ice-cold PBS.

### *Legionella pneumophila* infection

The wildtype *Legionella pneumophila* strain CR39 was cultured in ACES-buffered yeast extract broth supplemented with FeNO_3_ and cysteine at 37°C, as described [18]. Post-exponential-phase cultures of *L. pneumophila* were washed in PBS, resuspended in tissue culture media and added to adherent RAW264.7 cells in 10-cm culture dishes at an MOI of 0.1. Cells were harvested at 48 hpi and lysed in TRIzol.

### *Mycobacterial* infection

*Mycobacterium marinum* strain M [19] was cultured from stocks in 25-ml flasks with 10 ml of complete 7H9 medium for 36–48 h, incubated at 33°C/5%CO_2_. Mycobacterial cultures were washed with PBS, resuspended in DMEM plus 10% FBS, prepared as a single cell *M. marinum* suspension by passage through a tuberculin syringe, counted and adjusted for the MOI of 10 used for the *in vitro* infections.

*Mycobacterium smegmatis* from stocks [20] was cultured in two 14-ml snap cap tubes with 5 ml of complete 7H9 medium for 24–36 h and incubated with agitation at 37°C. Then, a single cell suspension was prepared, counted and adjusted for an MOI of 10.

Frozen stocks of *Mycobacterium tuberculosis* were cultured in complete 7H9 medium at 37°C/5%CO_2_ until their concentration reached an OD of 1.3. Then, cultures were spun, washed twice with 50 ml of PBS, resuspended in 4 ml, sonicated to give a single cell suspension and then diluted to give an MOI of 10.

To measure *M. marinum* MM-H RNA expression after different periods of infection, 12-well plates were inoculated with one ml of a RAW264.7 cell suspension (3.5×10^5^ cells/ml). After 24 h, the cells were infected with 500 µl of a single cell *M. marinum* suspension (MOI of 10) and incubated at 33°C for 12 h. Then 500 µl of streptomycin (200 µg/ml) were added to all the infected wells. For the first time point (12 h), media were removed and 500 µl TRizol was added for 5 min. The lysate was then added to an Eppendorf tube and stored at −80°C. The rest of the wells were incubated at 33°C and harvested as described for the previous timepoint at 24, 48 and 72 h post infection.

For small RNA deep sequencing, two 100-mm dishes of RAW264.7 cells at 70% confluency were infected using a single cell suspension of either *M. marinum* or *M. smegmatis* at an MOI of 10. After 24 h, the medium was removed, 4 ml of TRizol were added and the cells lysed for 20 min at room temperature with rocking agitation. For *M. tuberculosis*, 2×10^7^ THP-1 cells treated with PMA were infected with *M. tuberculosis* at an MOI of 10.

For RISC immunoprecipitation assays, nine dishes of RAW264.7 cells were infected with *M. marinum* and another nine with *M. smegmatis*. Nine dishes of PMA-treated THP-1 cells were infected with *M. tuberculosis*. After 24 h of infection, all culture medium was removed, each plate was washed with 10 ml of PBS and the cells then treated with 1 ml of lysis buffer (see below) and incubated with rocking agitation. Cells were scraped and liquid and cell debris collected, centrifuged at 4°C for 15 min at 13,000 g and then filtered through cellulose acetate membrane micro-columns.

### *In vivo* infection

This study was carried out in strict accordance with the recommendations in the Guide for the Care and Use of Laboratory Animals of the National Institutes of Health. All animal studies were approved by the Institutional Animal Care and Use Committee (IACUC) of Duke University (protocol A065-13-03). Animals were observed daily by staff and weighed once weekly. Infected animals were euthanized if they had lost more than 15% of their peak body weight. Euthanasia was by isofluorane overdose, followed by organ removal.

Specific pathogen-free C57BL/6 mice were obtained from Jackson Laboratories (Bar Harbor, ME). For each experiment, a vial of stock bacilli (3.6×10^8^) was aerosolized as described previously [21]. The number of viable organisms in each organ sample was determined by plating serial dilutions of the lung homogenates. Initial infection dose (determined by plating lung tissue 24 h post-infection) was 250 CFU and CFU at harvest (in other mice infected at the same time) was 4.3×10^5^±SD 1.9×10^5^. Lung tissue for RNA analysis was homogenized in 4.5 ml RNA*later* (Life Technologies) and then a 3× volume of TRIzol was added for RNA extraction.

### Deep sequencing

Total RNA was extracted using TRIzol (Life Technologies) and sequentially ligated to 3′ and 5′ adapters and then reverse-transcribed using a TruSeq Small RNA Sample Prep Kit (Illumina) and SSIII (Life Technologies) [22]. Total length of the adapters used was 125 nt. cDNAs were amplified by PCR. The number of PCR cycles was determined by pilot experiments to find a point at which amplicons showed logarithmic amplification. PCR products between 140 bp and 170 bp in length, which corresponds to original RNAs of between 15 and 45 nt, were then isolated by excision from a polyacrylamide gel. Deep-sequencing of the PCR products was performed using a HiSeq2500 machine (Illumina) for 50 base pair single read.

For RNA immunoprecipitation (RIP), cells were collected and lysed using lysis buffer (50 mM HEPES, 150 mM KCl, 2 mM EDTA, 1 mM NaF, 0.5% NP40, 0.5 mM DTT). Lysates were incubated with Dynabeads Protein G (Life Technologies) conjugated with an Ago-specific monoclonal antibody (ab57113, Abcam) [23]. The beads were repeatedly washed with PBS and then TRIzol was added to extract bound RNA, which was subjected to small RNA deep-sequencing as described above.

### Bioinformatics

Sequence reads were mapped to bacterial genomes (GenBank accession number: AM884176 for *C. trachomatis*, NC_002942 for *L. pneumophila*, NC_010612 for *M. marinum*, NC_008596 for *M. smegmatis* and AL123456 for *M. tuberculosis*) and the host genome (hg19 for human genome and mm9 for mouse genome) by Bowtie and analyzed by SAMtools and DARIO [24], [25]. RNA secondary structures were predicted by mfold [26]. Sequence data are available at the Sequence Read Archive, http://www.ncbi.nlm.nih.gov/sra (SRP042180).

### qRT-PCR

To confirm expression of a bacterial small RNA, total RNA was extracted with TRIzol, then reverse transcribed to cDNA and quantified by qPCR using a Custom TaqMan small RNA Assay (Life Technologies).

### Luciferase assay

Oligonucleotides bearing two copies of a perfect target sequence for the putative *M. marinum* miRNA MM-H were cloned into the 3′ UTR of the RLuc gene in a lentiviral vector. Another lentiviral vector, which contains the firefly luciferase (FLuc) gene, was used as a transduction control, as described previously [27]. Packaged virus was transduced into RAW264.7 cells 12 h after infection with *M. marinum*. Dual luciferase assays were performed 24 h after transduction (i.e., 36 h after infection with *M. marinum*) using a Dual-Luciferase Reporter Assay System (Promega), according to the manufacturer's protocol.

The potential pre-miRNA sequence for MM-H was cloned into the pSUPER vector where it is expressed under the control of an H1 pol III promoter [28]. The two 3′ C residues in this pre-miRNA sequence were mutated to five successive T residues in order to terminate Pol III transcription after the second encoded U residue. An oligonucleotide bearing two perfect target sequences for MM-H was then cloned into the 3′ UTR of the RLuc indicator gene in the psiCHECK2 vector (Promega), which also encodes an FLuc gene as an internal control. The expression vector and luciferase reporter vector were co-transfected into 293T cells using polyethylenimine. Dual luciferase assays were then performed 24 h after transfection, as described above.

## Results

### *Chlamydia trachomatis*

**Figure 1:**
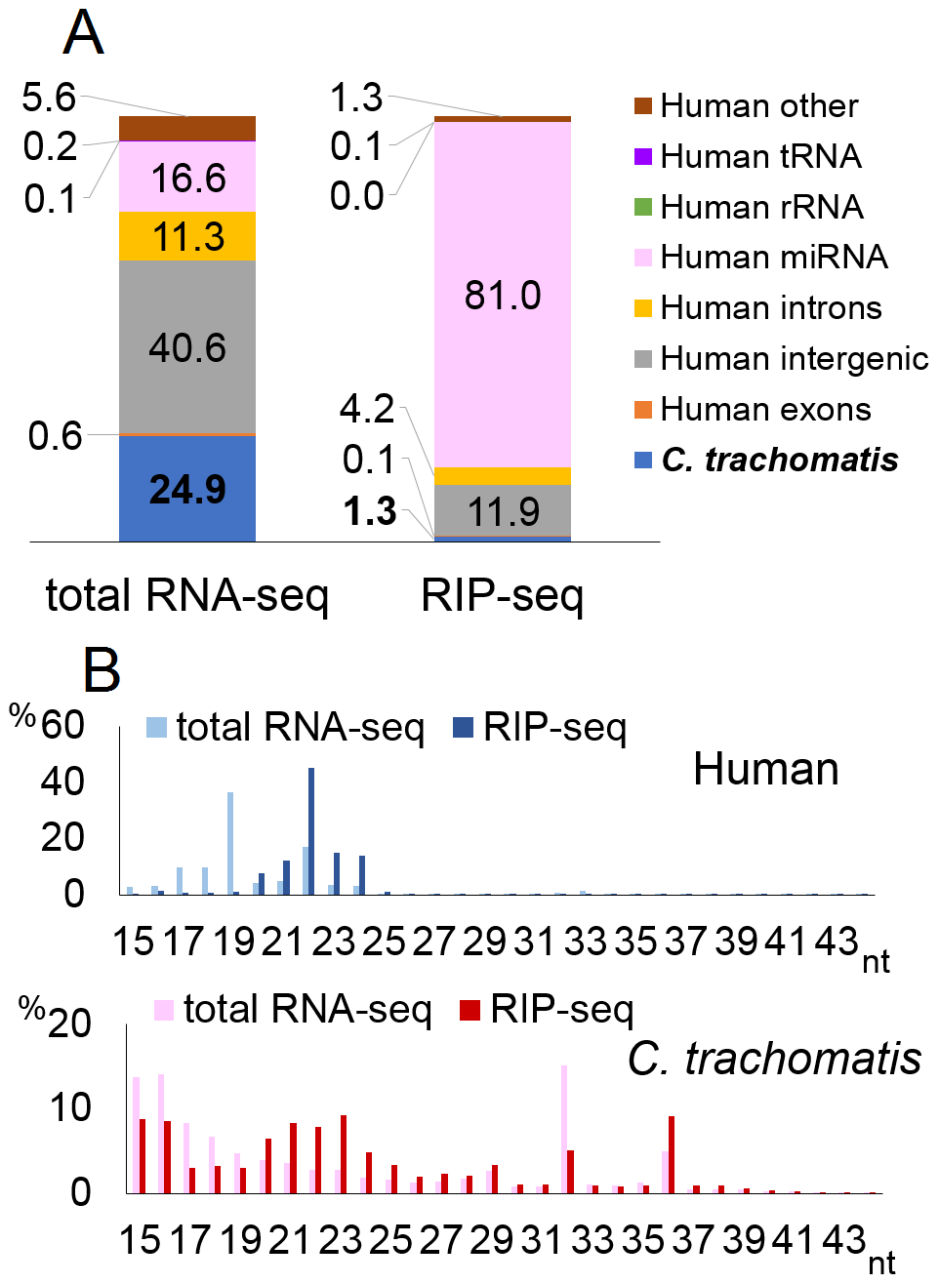
Deep-sequencing of small RNAs in *C. trachomatis*-infected cells. Results of deep-sequencing for *C. trachomatis*-infected HeLa cells. A) Gene annotation based on sequencing reads aligned to either human or bacterial genome. Values indicate the percentage of reads of each gene type in either the total or RISC IP from small RNA libraries. B) Length distribution of reads. The X-axis shows the length of small RNAs (nucleotide, nt) and the Y-axis shows the percentage of reads of each length in the total or RISC IP library.

*C. trachomatis* is an obligate intracellular bacterium that causes a major sexually transmitted disease as well as a potentially severe eye infection referred to as Chlamydia conjunctivitis or trachoma [29]. HeLa cells were infected with *C. trachomatis* at a multiplicity of infection (MOI) of 20 and total RNA isolated 42 h after infection. Deep-sequencing analysis of the small RNA transcriptome in the 15- to 43-nt size range (total RNA-seq) yielded ∼11 million reads which could be computationally aligned to either the human or bacterial genome, with 24.9% of reads derived from the bacterial genome (Fig. 1A). We next conducted RNA immunoprecipitation (RIP), using an antibody specific for the essential Argonaute (Ago) component of RISC [23], to enrich for small RNAs associated with the cellular RISC. Deep-sequencing analyses for these RISC-associated small RNAs (RIP-seq) yielded ∼18 million reads. When compared to the total RNA-seq data, the RIP-seq procedure substantially increased the percentage of reads that derived from known human miRNAs, from 16.6% to 81%. Consistent with their predominantly miRNA origin, human small RNAs recovered by RIP-seq showed a size of 22±2 nt, as predicted for authentic miRNAs (Fig. 1B). In contrast, reads derived from the bacterial genome declined from 24.9% of the observed reads in the total RNA-seq data to only 1.3% in the RIP-seq data (Fig. 1A) and these residual reads were not clustered at the 22±2-nt size (Fig. 1B).

Table 1 shows the 10 most abundant reads derived from the *C. trachomatis* genome, here referred to as CT-A through CT-J. Recovery of all of these 10 small RNAs was enhanced by RISC immunoprecipitation, which suggests these small RNAs are associated with RISC. The two most abundant bacterial small RNAs were derived from bacterial tRNAs (CT-A and CT-B) and were too large to be miRNAs, at 36 nt and 32 nt respectively. In fact, only CT-E, CT-H and CT-J showed a size typical for a miRNA. We modeled the RNA secondary structures adopted by these three small RNAs together with flanking bacterial genomic sequences, and CT-E and CT-H can indeed form possible RNA stem-loop structures (Table 1 and Fig. S1), as expected for pre-miRNAs. However, these small RNAs are derived from both the stem and loop regions of the predicted stem-loops. In contrast, canonical miRNAs are invariably entirely derived from one arm of the pre-miRNA stem [6], [7]. Moreover, it has been reported that, in order for a miRNA to be functionally relevant, it has to contribute >0.1% of the total miRNA pool in a cell [30]. Yet, none of the observed bacterial small RNAs account for more than 0.09% of the total miRNA pool (Table 1) and they are therefore unlikely to exert a significant phenotypic effect, even if they are loaded into RISC. Taken together, these data argue that *C. trachomatis* does not express miRNAs in infected human cells.

**Table 1:** Top 10 most prevalent small RNA reads of *C. trachomatis* origin.

ID	% of RISC-associated miRNA reads	5′ position in genome	Sense	Length (nt)	Origin	Stem-loop structure prediction	RIP-enrichment (fold) a
CT-A	0.087	55136	-	36	tRNA	NT b	15814.5
CT-B	0.063	364945	-	32	tRNA	NT	1.4
CT-C	0.047	416262	+	36	NCR	No	3.4
CT-D	0.032	385084	+	16	ORF	No	652.2
CT-E	0.028	605032	-	23	NCR	Noncanonical c	25.0
CT-F	0.021	834044	-	15	ORF	Noncanonical	394.8
CT-G	0.017	135763	+	29	NCR	No	7.5
CT-H	0.017	133471	-	20	NCR	Noncanonical	22.7
CT-I	0.010	229542	-	15	ORF (as)	No	8.6
CT-J	0.010	775954	-	23	NCR	No	19.7

a RIP-enrichment was calculated by percentage of reads in RIP-seq divided by that in total RNA-seq.

b NT, not tested. Secondary structure was not predicted because tRNAs are known to form a cloverleaf structure.

c Predicted stem-loop structure and origin of small RNA are depicted in Fig. S1.

NCR; non-coding region; ORF, open reading frame; as, anti-sense.

### *Legionella pneumophila*

**Figure 2:**
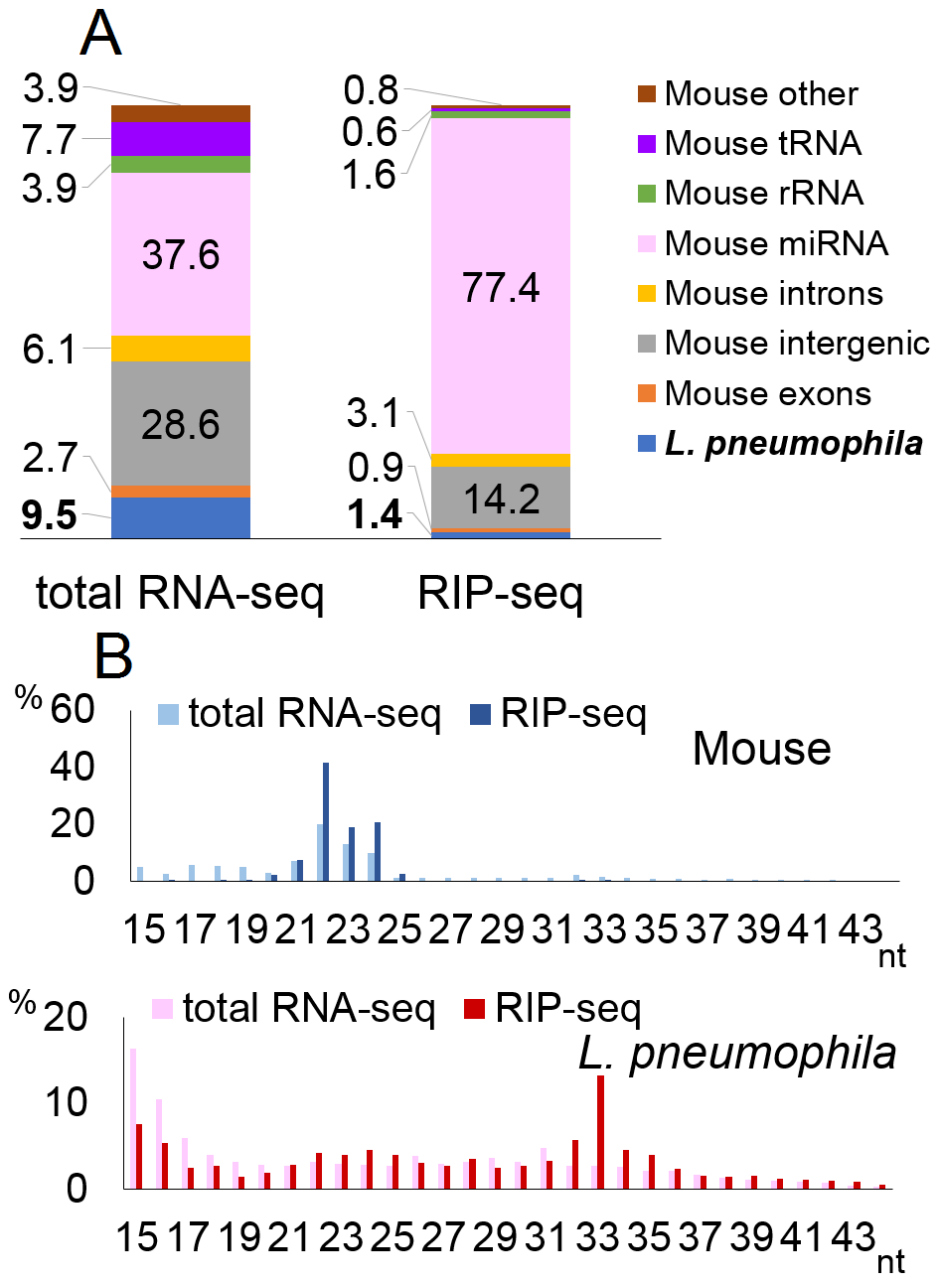
Deep-sequencing of small RNAs in *L. pneumophila*-infected cells. Results of deep-sequencing for *L. pneumophila*. The figures were generated as described in Fig. 1.

*L. pneumophila* is a Gram-negative bacterium commonly found in soil and fresh water sources where it resides and replicates inside free-living amoeba. Although *L. pneumophila* is primarily a pathogen of protozoa, aerosolized and inhaled bacteria can infect alveolar macrophages and cause opportunistic infections in humans and animal models [31]. The ability of *L. pneumophila* to successfully infect a broad spectrum of host species is likely founded in *Legionella*'s capacity to inject ∼300 distinct bacterial proteins into the host cytoplasm [32]. In order to determine whether *L. pneumophila* is also able to generate and transfer bacterial pre-miRNAs into host cells, we infected the murine macrophage-like cell line RAW264.7 with *L. pneumophila* at an MOI of 0.1 and isolated total RNA at 48 h post infection. Deep-sequencing analysis of the total small RNA population (15- to 43-nt size range) yielded ∼14 million reads which could be aligned to either the mouse or bacterial genome. Of these, 9.5% of were of *L. pneumophila* origin (Fig. 2A). Analysis of RISC-associated small RNAs, by immunoprecipitation using a pan-Ago antibody, yielded ∼20 million reads and, as expected, increased the percentage of reads that align to known murine miRNAs from 37.6% to 77.4% of the total small RNA population. Moreover, the murine reads obtained by RIP-seq were almost all in the expected 22±2-nt size window (Fig. 2B). In contrast, small RNA reads derived from the *L. pneumophila* genome decreased from 9.5% of the total small RNA reads to 1.4% of the RISC-associated small RNA reads (Fig. 2A) and were not clustered at the predicted 22±2-nt size expected for authentic miRNAs (Fig. 2B).

The 10 most abundant small RNA reads of *L. pneumophila* origin, termed LP-A through LP-J, are shown in Table 2 and all 10 were significantly increased in the RISC-associated small RNA library. However, 7 out of 10 were derived from bacterial tRNAs (Table 2). Of the three remaining reads, LP-D, together with flanking bacterial genomic sequences, is predicted to have the potential to fold into an RNA stem-loop structure (Fig. S2). Yet, the length of this small RNA, at 15 nt, is too short for an authentic miRNA [6] and all 10 of the predominant *L. pneumophila* small RNAs not only lie outside the expected size range for a functional miRNA but are also present at levels too low to be functionally relevant [30] (Table 2). Taken together, these data argue that *L. pneumophila* does not express miRNAs of bacterial origin in infected murine cells.

**Table 2:** Top 10 most prevalent small RNA reads of *L. pneumophila* origin.

ID	% of RISC-associated miRNA reads	5′ position in genome	Sense	Length (nt)	Origin	Stem-loop structure prediction	RIP-enrichment (fold)
LP-A	0.077	2099390	-	33	tRNA	NT	1261.9
LP-B	0.076	2099258	-	33	tRNA	NT	1157.8
LP-C	0.021	573714	+	18	ORF	Noncanonical a	1087.5
LP-D	0.020	771578	+	15	ORF (as)	Noncanonical	34.5
LP-E	0.018	2154466	+	32	tRNA	NT	977.0
LP-F	0.017	77551	-	32	tRNA	NT	7269.2
LP-G	0.014	2154466	+	33	tRNA	NT	344.4
LP-H	0.013	77548	-	35	tRNA	NT	1846.1
LP-I	0.009	2667931	+	33	tRNA (as)	NT	1025.4
LP-J	0.008	2099257	-	34	tRNA	NT	49.3

a Predicted stem-loop structure and origin of small RNA are depicted in Fig. S2. as, anti-sense.

### *Mycobacterium marinum*

**Figure 3:**
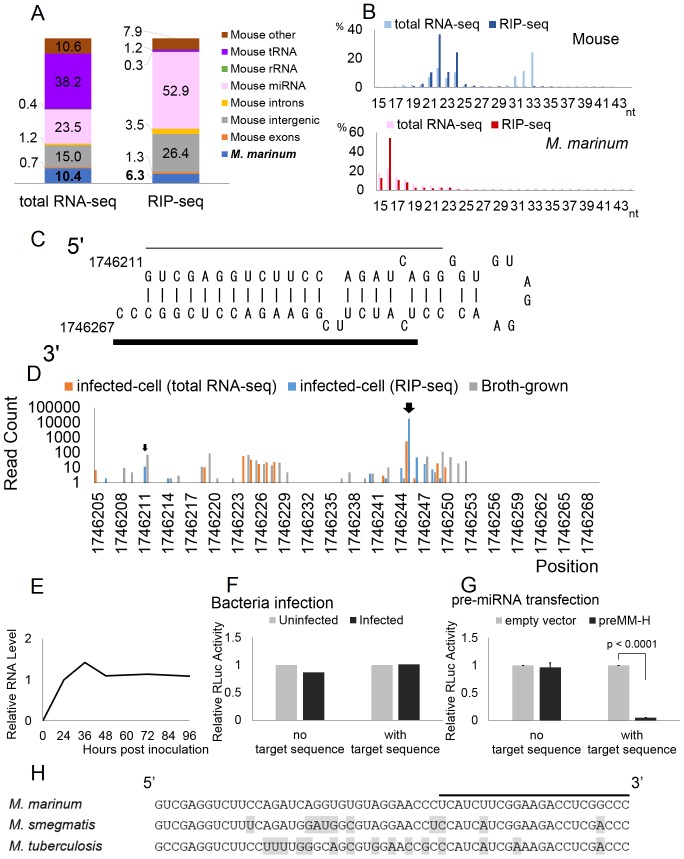
Deep-sequencing of small RNAs in *M. marinum* infected cells and characteristics of the MM-H miRNA candidate. A and B) Results of deep-sequencing for *M. marinum*. The figures were generated as described in Fig. 1. C) Predicted RNA secondary structure of a possible MM-H precursor, including flanking sequences. The bold line indicates the putative mature miRNA and the thin line indicates a possible passenger strand. D) 5′ end starting positions of small RNAs from the structure shown in panel a, found in infected cell lines (total RNA-seq and RIP-seq) and in broth-grown bacteria. The Y-axis shows the absolute read count of each small RNA. Large and small arrows indicate the starting positions of the putative mature miRNA and possible passenger strand shown in panel A. E) Time-course analysis of the expression level of the MM-H RNA measured by qRT-PCR. Relative expression levels relative to the 24-h time point were normalized to the host cell U6 RNA level are indicated. Data shown represent the average of two experiments. F) Inhibitory activity of the putative *M. marinum* MM-H miRNA. An RLuc-based indicator, with or without two copies of a perfectly complementary target sequence for the putative MM-H miRNA inserted into the 3′ UTR, was transduced into RAW264.7 cells infected or uninfected with *M. marinum*. Relative RLuc activity was measured at 24 h post-transduction and then normalized to uninfected control and to the FLuc internal control, which is present in a second lentiviral vector transduced simultaneously. A representative experiment is shown. G) Similar to panel F, except in this case a Pol III-based expression vector encoding the putative MM-H pre-miRNA shown in panel C was co-transfected into 293T cells along with an RLuc-based MM-H indicator plasmid and an FLuc-based internal control. Average of three experiments with SD indicated. H) Sequence homology of the MM-H region in various mycobacteria. The predominant *M. marinum* MM-H sequence is indicated by a bold line. Nucleotides that differ from *M. marinum* are highlighted.

*M. marinum*, a mycobacterium that is a natural intracellular pathogen of ectotherms [33], is the closest animal relative of the human *Mycobacterium tuberculosis* complex [19]. Mouse RAW264.7 cells were infected with *M. marinum* at an MOI of 10 and total RNA isolated 24 h after infection. Deep-sequencing analysis of the total small RNA population yielded ∼9 million reads which could be aligned to either the mouse or *M. marinum* genome. Of these, 10.4% were of bacterial origin (Fig. 3A). Deep sequencing of RISC-associated small RNAs by Pan-Ago RIP-seq yielded ∼16 million reads and 6.3% of these were of bacterial origin. Unlike the murine RISC-associated small RNA reads, which showed the expected 22±2-nt size, we did not observe a significant enrichment of bacterial reads of around 22 nt, and these instead clustered at <18 nt (Fig. 3B).

The 10 most common small RNA reads of *M. marinum* origin, termed MM-A to MM-J, are listed in Table 3. Among these, MM-H looked like a promising miRNA candidate. This small RNA is primarily 23 nt in length and is derived from a pre-miRNA-like stem-loop structure of canonical appearance, derived from a bacterial non-coding region, in which the MM-H sequence occupies one side of the predicted stem (Table 3 and Fig. 3C). Moreover, deep sequencing identified a small number of reads derived from the opposite side of the stem and offset by 2 nt at both 3′ ends, as expected for a miRNA passenger strand [34], [35] (Fig. 3C and Table S1). Also, the MM-H small RNA possessed a highly discrete 5′ end yet showed length variation at the 3′ end (Table S1), as would be predicted for an authentic miRNA, where the seed sequence located at the 5′ end is the primary determinant of mRNA target recognition [6]. This finding, which is unique to MM-H among all the various bacterial small RNAs described in this report, strongly suggests that the 5′ end of the MM-H small RNA results from a specific processing step rather than from random RNA degradation.

**Table 3:** Top 10 small RNA reads of *M. marinum*.

ID	% of RISC-associated miRNA reads	5′ position in genome	Sense	Length (nt)	Origin	Stem-loop structure prediction	RIP-enrichment (fold)
MM-A	4.56	45371	-	16	ORF (as)	Noncanonical a	4.5
MM-B	1.01	5093409	+	15	ORF	Noncanonical	1.7
MM-C	0.67	1488839	-	17	ORF (as)	No	2.6
MM-D	0.58	5738697	-	16	ORF	No	7.0
MM-E	0.30	1691734	-	18	ORF	Noncanonical	16.1
MM-F	0.19	962428	+	16	ORF (as)	Noncanonical	47.1
MM-G	0.18	1488839	-	16	ORF (as)	No	4.6
MM-H	0.18	1746245	+	23	NCR	Canonical b	17.8
MM-I	0.17	1706825	-	16	ORF (as)	Noncanonical	0.2
MM-J	0.17	4198905	-	18	ORF (as)	Noncanonical	10.2

a The predicted stem-loop structure and origin of several small RNAs is depicted in Fig. S3.

b Predicted stem-loop structure and origin of this small RNA is depicted in Fig. 3C.

*M. marinum* is a facultative intracellular parasite that is able to survive both inside or outside host cells and we wondered whether production of the MM-H small RNA, and particularly the precise definition of the 5′ end of MM-H, might be dependent on the mammalian cell miRNA processing machinery. We therefore conducted small RNA deep-sequencing analyses of broth-grown *M. marinum*. A limited number of small RNAs derived from this predicted bacterial RNA stem-loop were indeed recovered from the broth-grown bacteria. However, these small RNAs clearly did not possess a discrete 5′ end, as seen upon RIP-seq of RISC-associated bacterial small RNAs in mouse cells infected with *M. marinum* and no reads sharing the same 5′ end as the dominant intracellular form of MM-H were recovered (Fig. 3D). This result indicates that the MM-H bacterial small RNA requires the host cell RNA processing machinery for its biogenesis.

While quantitative RT-PCR (qRT-PCR) with MM-H-specific primers and probes confirmed MM-H expression by 24 h after infection of RAW264.7 cells by *M. marinum*, the level of expression did not increase further over the next 72 h (Fig. 3E). We next asked if MM-H was able to repress mRNA function using a Renilla luciferase (RLuc)-based indicator construct bearing two perfectly complementary target sites for MM-H in the RLuc 3′ UTR. This RLuc indicator cassette was transduced into RAW264.7 cells, either infected or uninfected with *M. marinum*, using a lentiviral vector and RLuc activity measured 24 h after transduction [27]. However, bacterial infection did not detectably suppress expression of the RLuc reporter gene (Fig. 3F).

We considered the possibility that the level of expression of MM-H in infected RAW264.7 cells, at ∼0.18% of the RISC-associated small RNA pool, might be too low to be active in gene repression (Table 3) [30]. To test this idea, we co-transfected 293T cells with the MM-H RLuc reporter vector together with a second vector in which the MM-H RNA, and flanking bacterial sequences sufficient to form a pre-miRNA-like stem-loop (Fig. 3C), were transcribed from a human Pol III promoter [28]. Exogenous overexpression of this putative bacterial pre-miRNA species in fact resulted in a dramatic reduction in the activity of the MM-H-specific RLuc reporter construct (Fig. 3G). Therefore, MM-H not only has many features typical of an authentic miRNA but also is able to effectively repress a target mRNA bearing perfectly complementary target sites when ectopically expressed in the form of the pre-miRNA shown in Fig. 3C.

### *Mycobacterium smegmatis*

**Figure 4:**
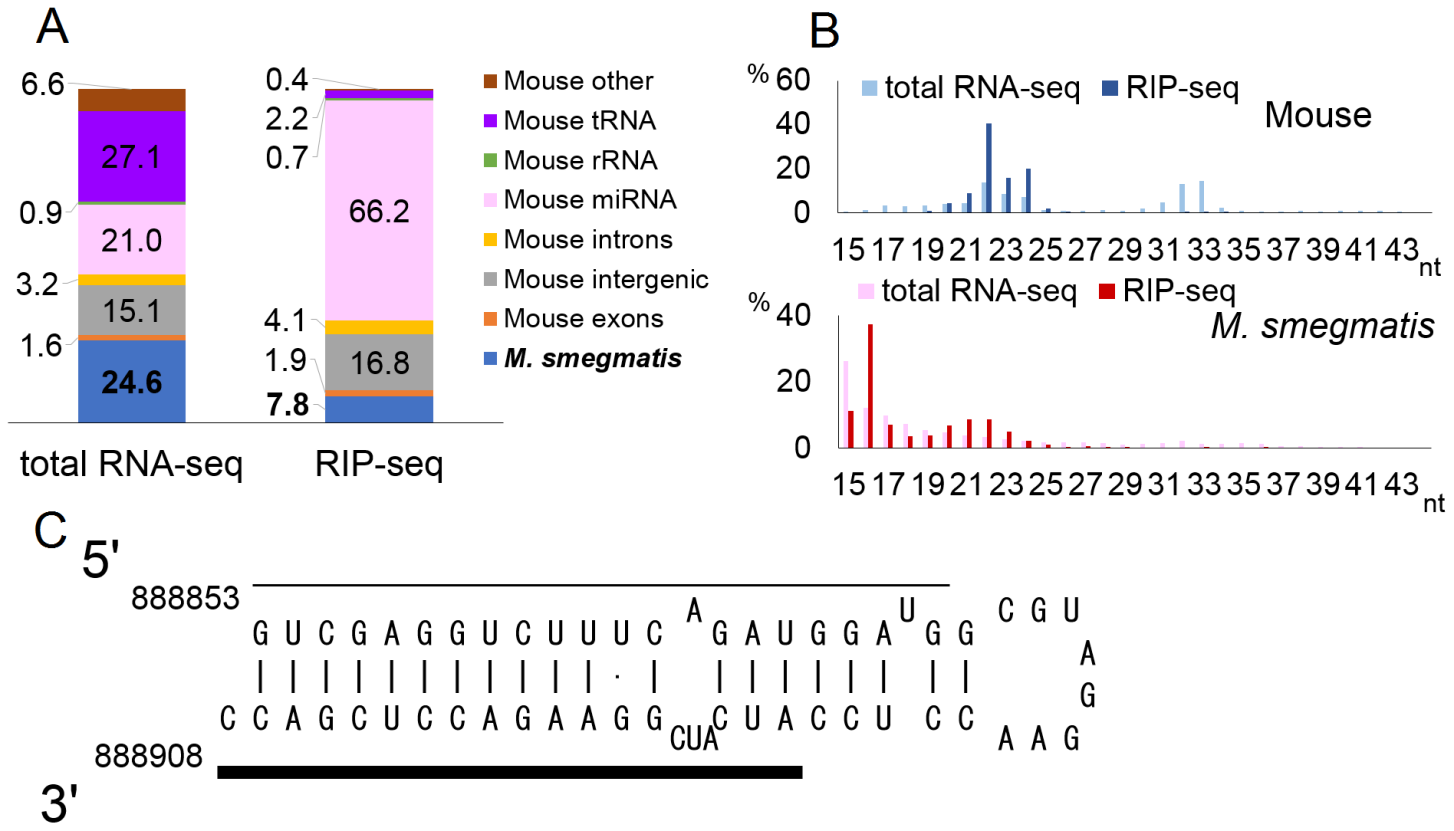
Deep-sequencing of small RNAs in *M. smegmatis*-infected cells. A and B) Results of small RNA deep-sequencing for *M. smegmatis*-infected RAW264.7 cells. These figures were generated as described in Fig. 1. C) Predicted RNA secondary structure of the MM-H homology region of *M. smegmatis*. Bold line indicates the putative mature miRNA and the thin line a possible passenger strand found by deep-sequencing.

*M. smegmatis* is a non-pathogenic mycobacterium defined as a fast-growing mycobacterial species [20]. Murine RAW264.7 cells were infected with *M. smegmatis*, at an MOI of 10 and total RNA isolated 36 h after infection. Deep-sequencing analysis of the total small RNA population yielded ∼15 million reads, of which 24.6% were of *M. smegmatis* origin (Fig. 4A). RIP-seq using an antibody specific for the Ago component of RISC resulted in ∼21 million computationally assignable reads and, as expected, enriched the proportion of reads that were of host cell miRNA origin from 21% to 66.2%. In contrast, the percentage of reads that map to the *M. smegmatis* genome declined from 24.6% to 7.8% of the total library. Again, murine small RNA reads in the RIP-seq library showed an obvious peak size of 22±2-nt (Fig. 4B), while the bacterial RISC-associated small RNAs were predominantly <19 nt in size. However, a secondary peak with a length of 21 to 24 nt could be observed for the *M. smegmatis*-derived small RNAs.

The top ten most prevalent *M. smegmati*s-derived small RNA species, designated MS-A through MS-J, were all enriched by RISC immunoprecipitation and several of these have the potential to form RNA stem-loop structure (Table 4). Yet, these bacterial small RNAs do not map to one arm of the predicted RNA stem-loops, as is invariably the case with authentic miRNAs [6], [7], and their length is consistently at or below 18 nt, too short for real miRNAs (Table 4 and Fig. S4).

**Table 4:** Top 10 small RNA reads of *M. smegmatis*.

ID	% of RISC-associated miRNA reads	5′ position in genome	Sense	Length (nt)	Origin	Stem-loop structure prediction	RIP-enrichment (fold)
MS-A	2.40	3325937	-	16	ORF (as)	No	25.8
MS-B	0.62	658512	-	16	ORF (as)	No	27.2
MS-C	0.61	5091600	+	15	ORF	Noncanonical a	27.0
MS-D	0.27	1454138	-	16	ORF (as)	Noncanonical	25.8
MS-E	0.24	3325937	-	17	ORF (as)	No	15.5
MS-F	0.16	4060958	-	16	ORF (as)	Noncanonical	40.8
MS-G	0.09	4987681	-	16	ORF	Noncanonical	174.5
MS-H	0.08	6366753	-	16	ORF (as)	Noncanonical	43.3
MS-I	0.08	4998098	-	18	ORF	Noncanonical	56.0
MS-J	0.07	4048594	+	17	pseudo gene (as)	Noncanonical	132.3

a The predicted stem-loop structures and origin of these small RNAs is depicted in Fig. S4.

We did not find *M. smegmatis* small RNA reads with homology to the *M. marinum* MM-H small RNA among the 10 most prevalent reads. However, the sequence of MM-H, and its flanking sequences, is relatively well conserved in several Mycobacterial species (Fig. 3H). Indeed, by deep-sequencing, we did find small RNAs from the homologous sequence in *M. smegmatis* derived from both the 5′ and 3′ arms of the cognate predicted RNA stem-loop structure (Fig. 4C and Table S2). However, the predicted seed sequence of MM-H [6] is not conserved in *M. smegmatis*, and the most prevalent small RNA that maps to this region in *M. smegmatis* was not enriched by RISC IP. Moreover, the expression level of this small RNA (889 reads for the most abundant sequence, which is equivalent to 0.004% of the total cellular small RNA population) is extremely low (Table S2).

### *Mycobacterium tuberculosis*

**Figure 5:**
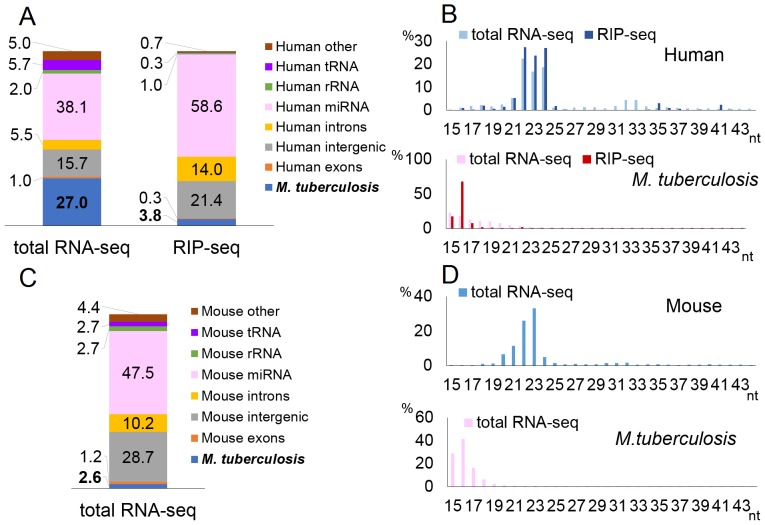
Deep-sequencing of small RNAs in *M. tuberculosis* infected cells and mice. These figures were generated as described in Fig. 1, for *in vitro* infection of THP-1 cells (A and B) and *in vivo* infection of mice (C and D).

To investigate *M. tuberculosis,* the mycobacterial pathogen of greatest importance to human health [36], we performed infections with *M. tuberculosis* of both human cell lines and of a mouse *in vivo* model. Human THP-1 cells were infected with *M. tuberculosis* at an MOI of 10 and total RNA isolated 36 h after infection. Total RNA-seq yielded ∼25 million reads that could be computationally assigned to either the human or *M. tuberculosis* genome, while RIP-seq using an antibody specific for cellular Ago proteins resulted in ∼31 million assignable reads. The percentage of reads that map to known human miRNAs increased from 38.1% of the total small RNA library to 58.6% of the RISC-associated small RNA library, as expected (Fig. 5A). Twenty-seven percent of reads mapped to the *M. tuberculosis* genome by total RNA-seq and this deceased sharply to 3.8% of the RISC-associated small RNAs. The length distribution of the human reads showed an obvious peak at around 22±2-nt, while the bacterial RISC-associated small RNAs clustered below 19 nt in size, too small for authentic miRNAs (Fig. 5B)

Of the 10 most prevalent bacterial small RNAs recovered from *M. tuberculosis* infected cells, here termed MT-A to MT-J, only one, MT-F, falls within the predicted length limits for an authentic miRNA (Table 5). This small RNA, together with its flanking genomic region, does have the potential to form an RNA stem-loop structure. However, the MT-F small RNA extends over the terminal loop into both arms of the stem, which is not consistent with its origin as a Dicer cleavage product (Fig. S5). Although the MM-H sequence from *M. marinum* is relatively well conserved in the *M. tuberculosis* genome (Fig. 3H), no small RNAs from this region were detected in *M. tuberculosis*-infected cells.

**Table 5:** Top 10 small RNA reads of *M. tuberculosis* in culture.

ID	% of RISC-associated miRNA reads	5′ position in genome	Sense	Length (nt)	Origin	Stem-loop structure prediction	RIP-enrichment (fold)
MT-A	3.66	1844427	-	16	ORF (as)	Noncanonical a	7.3
MT-B	0.56	1844427	-	15	ORF (as)	Noncanonical	5.9
MT-C	0.24	2040474	+	17	ORF	Noncanonical	5.1
MT-D	0.11	4362305	+	15	ORF (as)	Noncanonical	0.05
MT-E	0.10	3309774	-	15	ORF (as)	No	7.0
MT-F	0.07	3552591	+	22	NCR	Noncanonical	1.6
MT-G	0.04	3339219	-	16	ORF (as)	Noncanonical	8.3
MT-H	0.04	2565982	+	17	ORF	Noncanonical	0.2
MT-I	0.01	3335396	+	18	ORF (as)	Noncanonical	3.9
MT-J	0.03	863873	-	16	ORF (as)	No	8.0

a The predicted stem-loop structures and origin of these small RNAs is depicted in Fig. S5.

We also performed deep sequencing of small RNAs derived from an *M. tuberculosis in vivo* infection model [21] as a longer, persistent infection could result in the accumulation of bacterial small RNAs in infected cells. We infected mice with *M. tuberculosis* and isolated total RNA from the lungs 6 weeks after infection. Total RNA-seq for the sample yielded ∼21 million reads, of which 2.6% were of *M. tuberculosis* origin (Fig. 5C). Mouse reads peaked around 22±2-nt, while most bacterial reads were again shorter than 19 nt (Fig. 5D). Five of the 10 most prevalent bacterial reads recovered in this *in vivo* experiment overlapped with the top 10 reads observed in the infected THP-1 cells in culture (Tables 5 and 6). Yet, none of these has the characteristics expected for a miRNA; they are too short and the region from which small RNAs derive in the predicted RNA stem-loop structures is inconsistent with their origin as Dicer cleavage products [6] (Fig. S5). Small RNAs derived from the *M. tuberculosis* genomic sequence with homology to the *M. marinum* of MM-H small RNA (Fig. 3H) were not detected.

**Table 6:** Top 10 small RNA reads of *M. tuberculosis in vivo*.

	% of RISC-associated miRNA reads	5′ position in genome	Sense	Length (nt)	Origin	Stem-loop structure prediction	Correspondence to *in vitro* reads a
vMT-A	1.10	1844427	-	16	ORF (as)	Noncanonical b	MT-A
vMT-B	0.29	4362305	+	15	ORF (as)	Noncanonical	MT-D
vMT-C	0.22	2040474	+	17	ORF	Noncanonical	MT-C
vMT-D	0.18	1844427	-	15	ORF (as)	Noncanonical	MT-B
vMT-E	0.14	2739764	-	16	NCR	Noncanonical	-
vMT-F	0.11	777500	-	15	ORF	Noncanonical	-
vMT-G	0.06	777500	-	16	ORF	Noncanonical	-
vMT-H	0.06	936813	+	15	ORF (as)	Noncanonical	-
vMT-I	0.05	2565982	+	17	ORF	Noncanonical	MT-H

a Exact identity to a small RNA obtained *in vitro* is indicated.

b The predicted stem-loop structures and origin of these small RNAs is depicted in Fig. S5.

## Discussion

Previous work has identified a number of virally encoded miRNAs that enhance virus replication by down-regulating host cell mRNAs encoding proteins with antiviral potential [8], [9]. In at least one case, a viral miRNA has been shown to significantly enhance viral pathogenesis *in vivo* [37]. Similarly, a plant fungal pathogen has recently been shown to express miRNAs in infected plants that repress the host innate immune response and promote fungal pathogenicity [10]. We therefore hypothesized that intracellular bacteria, such as *C. trachomatis, L. pneumophila* or *Mycobacterium* spp., which are known to translocate bacterial proteins into the host cell [32], [38]–[40], might also encode miRNAs that could attenuate the ability of the host cell to resist bacterial invasion.

Because intracellular bacteria actively translocate microbial products into the host cell cytoplasm [32], we hypothesized that the most likely mechanism for bacterial miRNA production would involve the expression of a pre-miRNA-like hairpin RNA by the bacterium, either directly or as a result of RNA processing, that would be translocated into the cytoplasm and processed by the host cell Dicer protein to generate a miRNA duplex intermediate, one or both strands of which would then be loaded into RISC. In this way, the bacterium would be able to take advantage of the precise processing capacity of Dicer to generate a highly discrete 5′ end [6]. This would generate to a single miRNA seed sequence, located at positions 2 to 8 of the miRNA, and lead to targeting of a specific population of cellular mRNAs. Moreover, Dicer processing is thought to enhance miRNA loading into RISC [41].

If this hypothesis is correct, then small RNA sequencing of mammalian cells infected by intracellular bacterial pathogens should lead to the recovery of small RNAs of bacterial origin, ∼22±2 nt in length, that derive from one arm of a predicted ∼60-nt hairpin RNA. Ideally, we would also observe small RNA reads derived from the other arm of the same pre-miRNA hairpin, representing the passenger strand, offset by the predicted 2-nt 3′ overhangs [6]. The putative bacterial miRNA would be expected to have a discrete 5′ end, but not necessarily a discrete 3′ end, and to be associated with RISC. Finally, we would expect expression of this bacterial small RNA in infected cells to result in the repression of mRNAs bearing complementary target sequences, including mRNAs that are expressed from miRNA indicator constructs.

To address whether intracellular bacterial pathogens indeed express miRNAs in infected cells, we performed deep sequencing of small (15–43 nt long) RNAs expressed in mammalian cells infected by *C. trachomatis* and *L. pneumophila*, as well as three mycobacterial species, *M. marinum, M. smegmatis* and *M. tuberculosis.* All five of these bacteria gave rise to substantial levels of small bacterial RNAs, as determined by total small RNA deep sequencing, ranging from ∼27% of the total small RNA population in *M. tuberculosis* infected THP-1 cells to ∼9.5% of the small RNAs in *L. pneumophila-*infected RAW264.7 cells. However, for all five bacterial species the percentage of reads of bacterial origin dropped substantially when RISC-associated small RNAs were deep sequenced. This contrasts with authentic cellular miRNAs, which greatly increased as a percentage of the reads obtained upon sequencing of RISC-associated small RNAs (Figs. 1, 2, 3, 4, 5).

While the majority of small bacterial RNAs are therefore clearly not RISC-associated, we did identify several bacterial small RNAs that were enriched, sometimes to a remarkable extent, upon deep sequencing of RISC-associated—as opposed to total—small RNAs from infected cells (Tables 1, 2, 3, 4, 5, 6). However, these small RNAs are almost all unlikely to be authentic miRNAs. In particular, the large majority were not of the expected 22±2 nt size expected for authentic miRNAs, they were not predicted to fold into a canonical pre-miRNA-like stem-loop when flanking genomic RNAs were included (Figs. S1, S2, S3, S4, S5), they did not have a discrete 5′ end (data not shown) and they were generally expressed at levels (≤0.1% of the total miRNA pool) that are too low to be functionally relevant [6],[30].

Some interesting aspects of these data are nevertheless worth discussing. For example, it is striking that tRNA fragments were a major source for bacterial small RNAs in *C. trachomatis* and, especially, *L. pneumophila* but were not observed in any mycobacterial small RNA library (Tables 1, 2, 3, 4, 5, 6). RISC-associated human small RNAs of tRNA origin have been reported previously and were proposed to arise from Dicer processing of tRNA cloverleaf structures that had “collapsed” to form a simple RNA hairpin [42]. However, the recovered bacterial tRNA fragments were invariably ≥32 nt in length and are therefore too large to result from Dicer processing of bacterial tRNAs.

We note that some of the mycobacterial small RNAs recovered are actually expressed at quite high levels (Tables 3-6), thus potentially suggesting that they might be functionally relevant. We also note that several of the bacterial small RNAs that were recovered at high levels from *M. tuberculosis*-infected human THP-1 cells were also among the most highly expressed small bacterial RNAs recovered from the lungs of *M. tuberculosis*-infected mice (Tables 5 and 6), thus demonstrating a remarkable level of reproducibility and again suggesting a potential functional relevance. Despite these intriguing findings, the overall conclusion is that, with one single possible exception, none of the bacterial small RNAs reported here has the characteristics of an authentic miRNA. That exception is the *M. marinum* MM-H small RNA. In particular, we note that:

1. All the MM-H RNA reads derive from one arm of a predicted pre-miRNA-like hairpin (Fig. 3C and Table S1).
2. We obtained 12 reads that derive from the opposite side of the predicted MM-H stem-loop and that are offset by 2 nt from the MM-H small RNA, consistent with their origin as a miRNA passenger strand and strongly suggesting that MM-H is generated by Dicer cleavage of the proposed RNA hairpin (Fig. 3C and Table S1).
3. The 5′ end of MM-H is highly discrete when recovered from *M. marinum*-infected cells (Table S1) but was “ragged” when small RNAs from broth-grown *M. marinum* were sequenced (Fig. 3D). Indeed, no small RNAs with the same 5′ start site as the MM-H small RNA recovered from infected cells were identified in the broth-grown *M. marinum* culture. This again argues that a host cell-specific RNA processing factor, presumably Dicer, was required to generate MM-H in infected cells.
4. MM-H reads were enriched by 18-fold as a percentage of the total small RNA population in the RISC-associated versus total small RNA library, thus suggesting that the MM-H RNA is loaded into RISC.
5. The major MM-H variant represented ∼0.18% of the RISC-associated miRNA pool in infected cells (Table 3), thus suggesting a level of expression that at least has the potential to exert a phenotypic effect [30].

Despite this latter prediction, we were not, in fact, able to detect repression of an RLuc-based MM-H indicator plasmid in *M. marinum*-infected RAW264.7 cells (Fig. 3F). We considered that MM-H is actually expressed in RAW264.7 cells at a level that is very close to the threshold of 0.1% of the total miRNA pool below which miRNAs are invariably ineffective [30]. To test this idea, we therefore expressed the pre-miRNA-like RNA hairpin for MM-H (Fig. 3C) using an RNA Pol III-based expression vector and indeed saw a dramatic and specific inhibition of the same RLuc-based indicator plasmid (Fig. 3G). Therefore, it is clear that the pre-MM-H hairpin can indeed serve as a substrate for Dicer cleavage and that the mature MM-H small RNA has the potential to load into RISC and act as a repressor of target mRNA expression. However, it remains unclear whether MM-H is an authentic bacterial miRNA generated from a pre-miRNA hairpin that is secreted by *M. marinum* into the infected cell cytoplasm or simply a bacterial RNA fragment that is fortuitously generated by Dicer cleavage of a stable bacterial RNA hairpin that is inadvertently released into the cell cytoplasm from bacteria that have lysed.

In favor of the idea that MM-H is not a true bacterial miRNA are the inability to detect MM-H function in *M. marinum*-infected RAW264.7 cells (Fig. 3F) and the fact that MM-H is not conserved, in terms of sequence or expression, in two other related mycobacterial species, *M. smegmatis* and *M. tuberculosis* (Fig. 3H, Table S2). Nevertheless, it remains possible that MM-H may accumulate to higher, functionally relevant levels *in vivo* that greatly exceed the low level of MM-H expression seen in culture (Fig. 3E). It is worth noting that among the vacuole-bound pathogens analyzed in this report, the pathogenic mycobacteria are thought to be unique in their ability to escape into the cytoplasm [43]–[45], which might greatly facilitate the release of a pre-miRNA precursor into the cellular compartment where Dicer is active. The identification of at least one candidate bacterial miRNA suggests that other intracellular pathogens, e.g., *Shigella, Rickettsia, Salmonella* and *Listeria*, are also worth examining for the potential expression of bacterial miRNAs.

## Supporting Information

Figure S1 **Predicted secondary structures for small RNA of** ***C. trachomatis*** **.** Predicted RNA secondary structure and origin of small RNAs of *C. trachomatis* listed in Table 1. The structures were predicted by mfold. The large black arrow and the white arrow indicate the 5′ and 3′ ends of the recovered small RNA, respectively. Small arrows indicate possible passenger strands, if detected.
(PDF)

Figure S2 **Predicted secondary structure for small RNAs of** ***L. pneumophila*** **.** Predicted secondary structures and origin of small RNAs of *L. pneumophila*, listed in Table 2. The structures were predicted by mfold. The large black arrow and the white arrow indicate the 5′ and 3′ ends of the recovered small RNA, respectively.
(PDF)

Figure S3 **Predicted secondary structure for small RNAs of** ***M. marinum*** **.** Predicted secondary structure and origin of several small RNAs of *M. marinum*, listed in Table 3. The large black arrow and the white arrow indicate the 5′ and 3′ ends of the recovered small RNA, respectively. Small arrows indicate possible passenger strands, if detected.
(PDF)

Figure S4 **Predicted secondary structure for small RNAs of** ***M. smegmatis*** **.** Predicted secondary structures and origin of small RNAs of *M. smegmatis*, listed in Table 4. The large black arrow and the white arrow indicate the 5′ and 3′ ends of the recovered small RNA, respectively. Small arrows indicate possible passenger strands, if detected.
(PDF)

Figure S5 **Predicted secondary structures for small RNAs of** ***M. tuberculosis*** **.** Predicted secondary structures and origin of small RNAs of *M. tuberculosis*, listed in Tables 5 and 6. The large black arrow and the white arrow indicate the 5′ and 3′ ends of the recovered small RNA, respectively.
(PDF)

Table S1 **Small RNAs derived from the** ***M. marinum*** **MM-H stem-loop structure.**
(PDF)

Table S2 **Small RNAs derived from the MM-H homology region of** ***M. smegmatis*** **.**
(PDF)

## References

[1] Fire A, Xu S, Montgomery MK, Kostas SA, Driver SE, et al (1998) Potent and specific genetic interference by double-stranded RNA in Caenorhabditis elegans. Nature 391: 806–811. 9486653 10.1038/35888

[2] Elbashir SM, Lendeckel W, Tuschl T (2001) RNA interference is mediated by 21- and 22-nucleotide RNAs. Genes Dev 15: 188–200. 11157775 10.1101/gad.862301 PMC312613

[3] Martinez J, Patkaniowska A, Urlaub H, Luhrmann R, Tuschl T (2002) Single-stranded antisense siRNAs guide target RNA cleavage in RNAi. Cell 110: 563–574. 12230974 10.1016/s0092-8674(02)00908-x

[4] Bernstein E, Caudy AA, Hammond SM, Hannon GJ (2001) Role for a bidentate ribonuclease in the initiation step of RNA interference. Nature 409: 363–366. 11201747 10.1038/35053110

[5] Hammond SM, Bernstein E, Beach D, Hannon GJ (2000) An RNA-directed nuclease mediates post-transcriptional gene silencing in Drosophila cells. Nature 404: 293–296. 10749213 10.1038/35005107

[6] Bartel DP (2009) MicroRNAs: target recognition and regulatory functions. Cell 136: 215–233. 19167326 10.1016/j.cell.2009.01.002 PMC3794896

[7] Cullen BR (2004) Transcription and processing of human microRNA precursors. Mol Cell 16: 861–865. 15610730 10.1016/j.molcel.2004.12.002

[8] Cullen BR (2013) MicroRNAs as mediators of viral evasion of the immune system. Nat Immunol 14: 205–210. 23416678 10.1038/ni.2537 PMC3642974

[9] Grundhoff A, Sullivan CS (2011) Virus-encoded microRNAs. Virology 411: 325–343. 21277611 10.1016/j.virol.2011.01.002 PMC3052296

[10] Weiberg A, Wang M, Lin FM, Zhao H, Zhang Z, et al (2013) Fungal small RNAs suppress plant immunity by hijacking host RNA interference pathways. Science 342: 118–123. 24092744 10.1126/science.1239705 PMC4096153

[11] Hoe CH, Raabe CA, Rozhdestvensky TS, Tang TH (2013) Bacterial sRNAs: regulation in stress. Int J Med Microbiol 303: 217–229. 23660175 10.1016/j.ijmm.2013.04.002

[12] Lalaouna D, Simoneau-Roy M, Lafontaine D, Masse E (2013) Regulatory RNAs and target mRNA decay in prokaryotes. Biochim Biophys Acta 1829: 742–747. 23500183 10.1016/j.bbagrm.2013.02.013

[13] Driessen AJ, Nouwen N (2008) Protein translocation across the bacterial cytoplasmic membrane. Annu Rev Biochem 77: 643–667. 18078384 10.1146/annurev.biochem.77.061606.160747

[14] Garneau JE, Dupuis ME, Villion M, Romero DA, Barrangou R, et al (2010) The CRISPR/Cas bacterial immune system cleaves bacteriophage and plasmid DNA. Nature 468: 67–71. 21048762 10.1038/nature09523

[15] Marraffini LA, Sontheimer EJ (2010) CRISPR interference: RNA-directed adaptive immunity in bacteria and archaea. Nat Rev Genet 11: 181–190. 20125085 10.1038/nrg2749 PMC2928866

[16] Gripenland J, Netterling S, Loh E, Tiensuu T, Toledo-Arana A, et al (2010) RNAs: regulators of bacterial virulence. Nat Rev Microbiol 8: 857–866. 21079634 10.1038/nrmicro2457

[17] Sampson TR, Saroj SD, Llewellyn AC, Tzeng YL, Weiss DS (2013) A CRISPR/Cas system mediates bacterial innate immune evasion and virulence. Nature 497: 254–257. 23584588 10.1038/nature12048 PMC3651764

[18] Coers J, Monahan C, Roy CR (1999) Modulation of phagosome biogenesis by Legionella pneumophila creates an organelle permissive for intracellular growth. Nat Cell Biol 1: 451–453. 10559990 10.1038/15687

[19] Stinear TP, Seemann T, Harrison PF, Jenkin GA, Davies JK, et al (2008) Insights from the complete genome sequence of Mycobacterium marinum on the evolution of Mycobacterium tuberculosis. Genome Res 18: 729–741. 18403782 10.1101/gr.075069.107 PMC2336800

[20] Shepard CC (1957) Growth characteristics of tubercle bacilli and certain other mycobacteria in HeLa cells. J Exp Med 105: 39–48. 13385405 10.1084/jem.105.1.39 PMC2136667

[21] Smith KL, Saini D, Bardarov S, Larsen M, Frothingham R, et al (2014) Reduced virulence of an extensively drug-resistant outbreak strain of Mycobacterium tuberculosis in a murine model. PLoS One 9: e94953. 24733050 10.1371/journal.pone.0094953 PMC3986381

[22] Skalsky RL, Corcoran DL, Gottwein E, Frank CL, Kang D, et al (2012) The viral and cellular microRNA targetome in lymphoblastoid cell lines. PLoS Pathog 8: e1002484. 22291592 10.1371/journal.ppat.1002484 PMC3266933

[23] Flores O, Nakayama S, Whisnant AW, Javanbakht H, Cullen BR, et al (2013) Mutational inactivation of herpes simplex virus 1 microRNAs identifies viral mRNA targets and reveals phenotypic effects in culture. J Virol 87: 6589–6603. 23536669 10.1128/JVI.00504-13 PMC3676078

[24] Langmead B, Trapnell C, Pop M, Salzberg SL (2009) Ultrafast and memory-efficient alignment of short DNA sequences to the human genome. Genome Biol 10: R25. 19261174 10.1186/gb-2009-10-3-r25 PMC2690996

[25] Fasold M, Langenberger D, Binder H, Stadler PF, Hoffmann S (2011) DARIO: a ncRNA detection and analysis tool for next-generation sequencing experiments. Nucleic Acids Res 39: W112–117. 21622957 10.1093/nar/gkr357 PMC3125765

[26] Zuker M (2003) Mfold web server for nucleic acid folding and hybridization prediction. Nucleic Acids Res 31: 3406–3415. 12824337 10.1093/nar/gkg595 PMC169194

[27] Gottwein E, Cai X, Cullen BR (2006) A novel assay for viral microRNA function identifies a single nucleotide polymorphism that affects Drosha processing. J Virol 80: 5321–5326. 16699012 10.1128/JVI.02734-05 PMC1472151

[28] Brummelkamp TR, Bernards R, Agami R (2002) A system for stable expression of short interfering RNAs in mammalian cells. Science 296: 550–553. 11910072 10.1126/science.1068999

[29] Belland R, Ojcius DM, Byrne GI (2004) Chlamydia. Nat Rev Microbiol 2: 530–531. 15248311 10.1038/nrmicro931

[30] Mullokandov G, Baccarini A, Ruzo A, Jayaprakash AD, Tung N, et al (2012) High-throughput assessment of microRNA activity and function using microRNA sensor and decoy libraries. Nat Methods 9: 840–846. 22751203 10.1038/nmeth.2078 PMC3518396

[31] Friedman H, Yamamoto Y, Klein TW (2002) Legionella pneumophila pathogenesis and immunity. Semin Pediatr Infect Dis 13: 273–279. 12491233 10.1053/spid.2002.127206

[32] Isaac DT, Isberg R (2014) Master manipulators: an update on Legionella pneumophila Icm/Dot translocated substrates and their host targets. Future Microbiol 9: 343–359. 24762308 10.2217/fmb.13.162 PMC4148032

[33] Tobin DM, Ramakrishnan L (2008) Comparative pathogenesis of Mycobacterium marinum and Mycobacterium tuberculosis. Cell Microbiol 10: 1027–1039. 18298637 10.1111/j.1462-5822.2008.01133.x

[34] Umbach JL, Cullen BR (2010) In-depth analysis of Kaposi's sarcoma-associated herpesvirus microRNA expression provides insights into the mammalian microRNA-processing machinery. J Virol 84: 695–703. 19889781 10.1128/JVI.02013-09 PMC2798371

[35] Okamura K, Phillips MD, Tyler DM, Duan H, Chou YT, et al (2008) The regulatory activity of microRNA* species has substantial influence on microRNA and 3′ UTR evolution. Nat Struct Mol Biol 15: 354–363. 18376413 10.1038/nsmb.1409 PMC2698667

[36] Philips JA, Ernst JD (2012) Tuberculosis pathogenesis and immunity. Annu Rev Pathol 7: 353–384. 22054143 10.1146/annurev-pathol-011811-132458

[37] Zhao Y, Xu H, Yao Y, Smith LP, Kgosana L, et al (2011) Critical role of the virus-encoded microRNA-155 ortholog in the induction of Marek's disease lymphomas. PLoS Pathog 7: e1001305. 21383974 10.1371/journal.ppat.1001305 PMC3044692

[38] Hubber A, Roy CR (2010) Modulation of host cell function by Legionella pneumophila type IV effectors. Annu Rev Cell Dev Biol 26: 261–283. 20929312 10.1146/annurev-cellbio-100109-104034

[39] Mueller KE, Plano GV, Fields KA (2014) New frontiers in type III secretion biology: the Chlamydia perspective. Infect Immun 82: 2–9. 24126521 10.1128/IAI.00917-13 PMC3911841

[40] van der Woude AD, Luirink J, Bitter W (2013) Getting across the cell envelope: mycobacterial protein secretion. Curr Top Microbiol Immunol 374: 109–134. 23239236 10.1007/82_2012_298

[41] Wang HW, Noland C, Siridechadilok B, Taylor DW, Ma E, et al (2009) Structural insights into RNA processing by the human RISC-loading complex. Nat Struct Mol Biol 16: 1148–1153. 19820710 10.1038/nsmb.1673 PMC2845538

[42] Babiarz JE, Ruby JG, Wang Y, Bartel DP, Blelloch R (2008) Mouse ES cells express endogenous shRNAs, siRNAs, and other Microprocessor-independent, Dicer-dependent small RNAs. Genes Dev 22: 2773–2785. 18923076 10.1101/gad.1705308 PMC2569885

[43] Stamm LM, Morisaki JH, Gao LY, Jeng RL, McDonald KL, et al (2003) Mycobacterium marinum escapes from phagosomes and is propelled by actin-based motility. J Exp Med 198: 1361–1368. 14597736 10.1084/jem.20031072 PMC2194249

[44] van der Wel N, Hava D, Houben D, Fluitsma D, van Zon M, et al (2007) M. tuberculosis and M. leprae translocate from the phagolysosome to the cytosol in myeloid cells. Cell 129: 1287–1298. 17604718 10.1016/j.cell.2007.05.059

[45] Watson RO, Manzanillo PS, Cox JS (2012) Extracellular M. tuberculosis DNA targets bacteria for autophagy by activating the host DNA-sensing pathway. Cell 150: 803–815. 22901810 10.1016/j.cell.2012.06.040 PMC3708656

