# Supplementary material for: Search for MicroRNAs Expressed by Intracellular Bacterial Pathogens in Infected Mammalian Cells
Source: PLoS One. 2014 Sep 3;9(9):e106434. doi: 10.1371/journal.pone.0106434 (PMC4153649; doi:10.1371/journal.pone.0106434)
Supplement: Figure S1 — Predicted secondary structures for small RNA of C. trachomatis . Predicted RNA secondary structure and origin of small RNAs of C. trachomatis listed in Table 1. The structures were predicted by mfold. The large black arrow and the white arrow indicate the 5′ and 3′ ends of the recovered small RNA, respectively. Small arrows indicate possible passenger strands, if detected. (PDF) [file pone.0106434.s001.pdf]

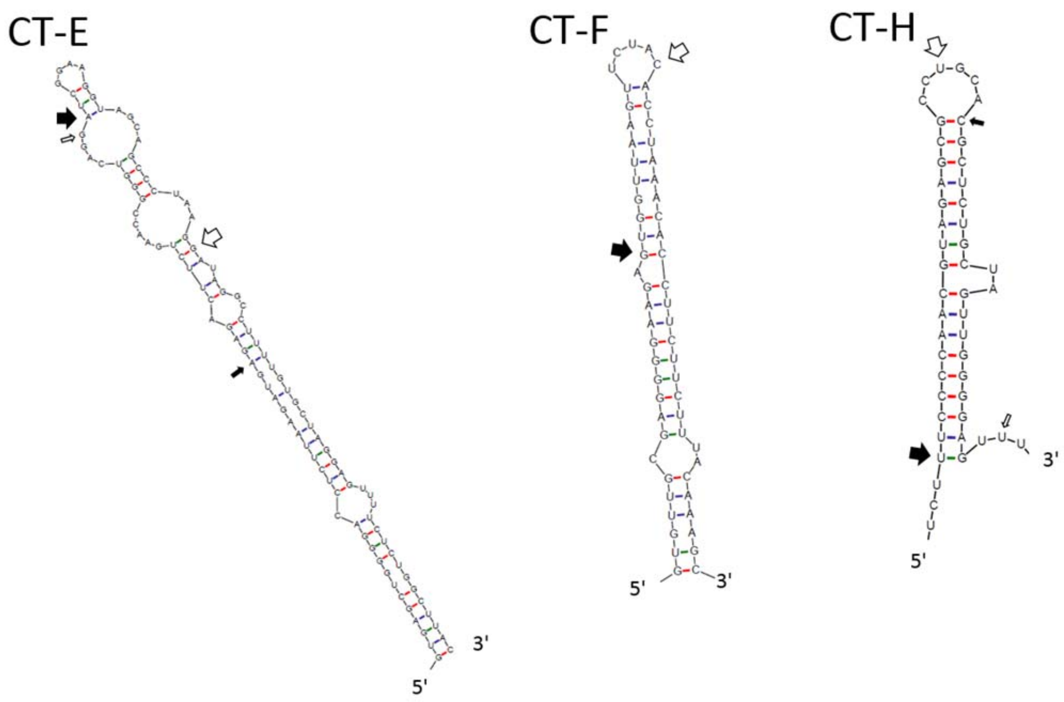

Figure S1. Predicted secondary structures for small RNA of *C. trachomatis*.

Predicted RNA secondary structure and origin of small RNAs of *C. trachomatis* listed in Table 1. The structures were predicted by mfold. The large black arrow and the white arrow indicate the 5’ and 3’ ends of the recovered small RNA, respectively. Small arrows indicate possible passenger strands, if detected.
