# Supplementary material for: Search for MicroRNAs Expressed by Intracellular Bacterial Pathogens in Infected Mammalian Cells
Source: PLoS One. 2014 Sep 3;9(9):e106434. doi: 10.1371/journal.pone.0106434 (PMC4153649; doi:10.1371/journal.pone.0106434)
Supplement: Figure S4 — Predicted secondary structure for small RNAs of M. smegmatis . Predicted secondary structures and origin of small RNAs of M. smegmatis, listed in Table 4. The large black arrow and the white arrow indicate the 5′ and 3′ ends of the recovered small RNA, respectively. Small arrows indicate possible passenger strands, if detected. (PDF) [file pone.0106434.s004.pdf]

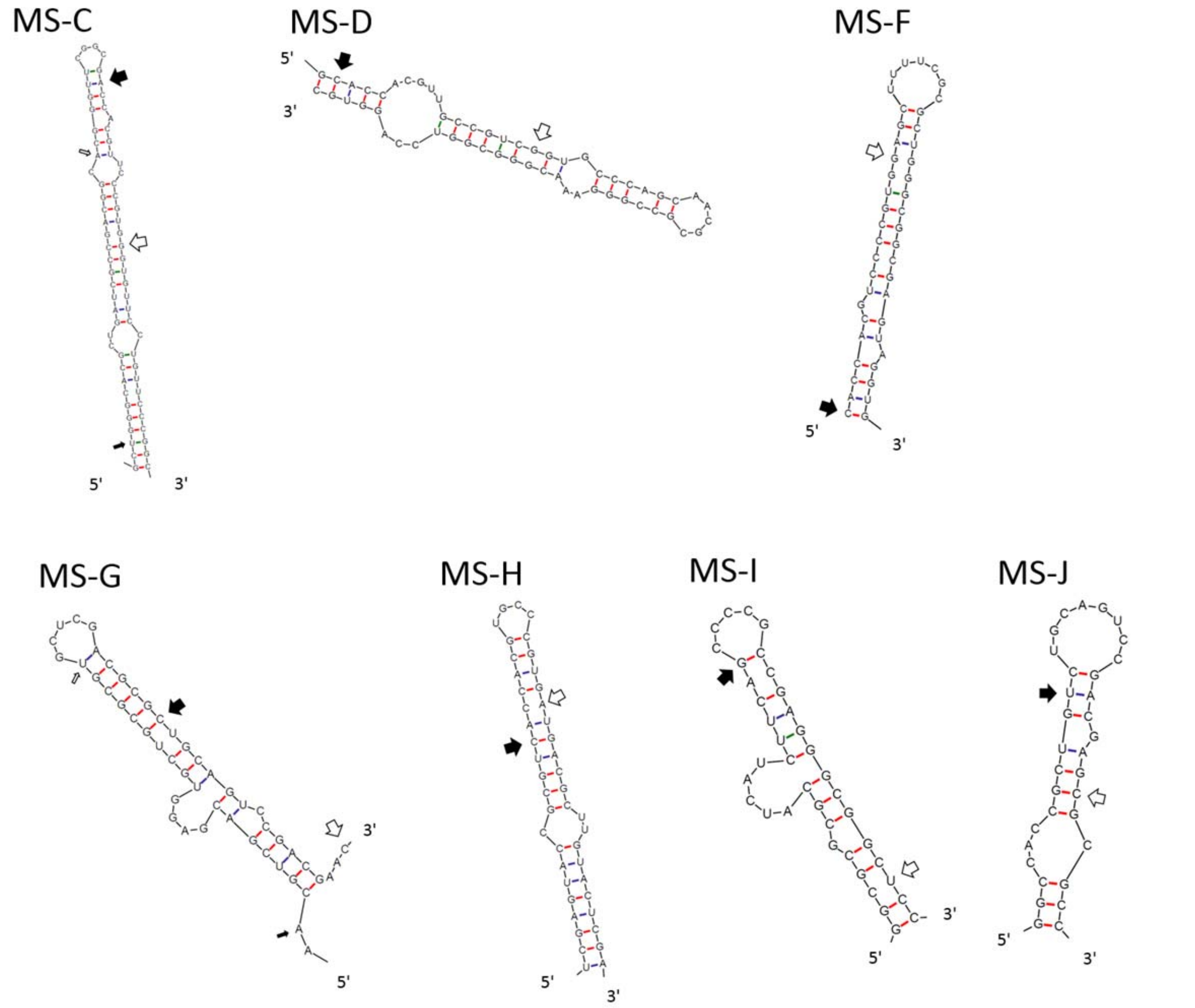

Figure S4. Predicted secondary structure for small RNAs of *M. smegmatis*.

Predicted secondary structures and origin of small RNAs of *M. smegmatis*, listed in Table 4. The large black arrow and the white arrow indicate the 5' and 3' ends of the recovered small RNA, respectively. Small arrows indicate possible passenger strands, if detected.
