# Supplementary material for: Search for MicroRNAs Expressed by Intracellular Bacterial Pathogens in Infected Mammalian Cells
Source: PLoS One. 2014 Sep 3;9(9):e106434. doi: 10.1371/journal.pone.0106434 (PMC4153649; doi:10.1371/journal.pone.0106434)
Supplement: Figure S5 — Predicted secondary structures for small RNAs of M. tuberculosis . Predicted secondary structures and origin of small RNAs of M. tuberculosis, listed in Tables 5 and 6. The large black arrow and the white arrow indicate the 5′ and 3′ ends of the recovered small RNA, respectively. (PDF) [file pone.0106434.s005.pdf]

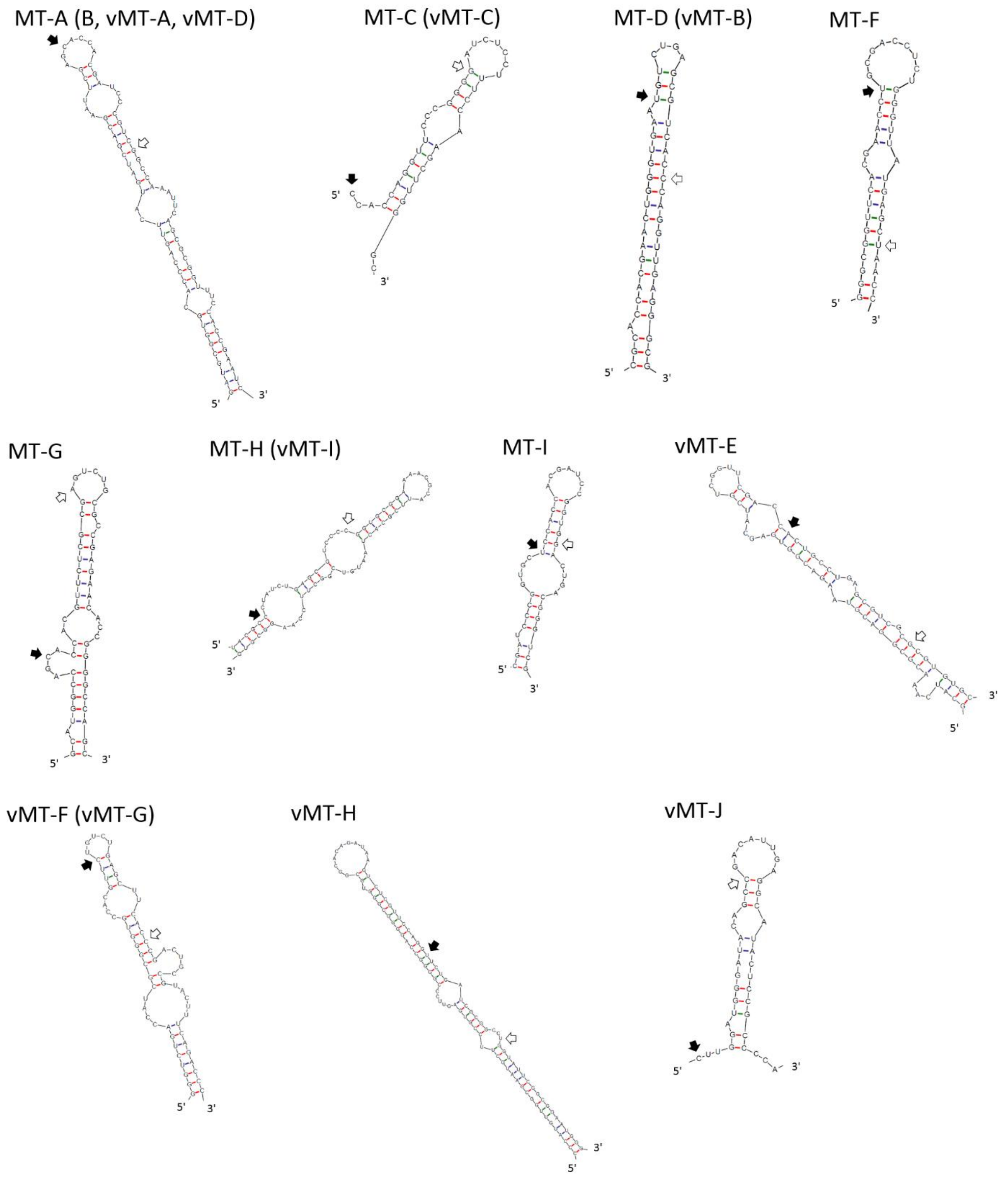

Figure S5. Predicted secondary structures for small RNAs of *M. tuberculosis*.

Predicted secondary structures and origin of small RNAs of *M. tuberculosis*, listed in Tables 5 and 6. The large black arrow and the white arrow indicate the 5’ and 3’ ends of the recovered small RNA, respectively.
