# Supplementary material for: Search for MicroRNAs Expressed by Intracellular Bacterial Pathogens in Infected Mammalian Cells
Source: PLoS One. 2014 Sep 3;9(9):e106434. doi: 10.1371/journal.pone.0106434 (PMC4153649; doi:10.1371/journal.pone.0106434)
Supplement: Table S1 — Small RNAs derived from the M. marinum MM-H stem-loop structure. (PDF) [file pone.0106434.s006.pdf]

Table S1. Small RNAs derived from the *M. marinum* MM-H stem-loop structure.

Strand	Position	Length	Sequence	Read count
5p	1746211-1746231	21	GUCGAGGUCUUCCAGAUCAGG	12
3p	1746245-1746265	21	UCAUCUUCGGAAGACCUCGGC	272
	1746245-1746266	22	UCAUCUUCGGAAGACCUCGGCC	1814
	**1746245-1746267**	**23**	UCAUCUUCGGAAGACCUCGGCCC	**16135 (0.18%)** [a]
	1746245-1746268	24	UCAUCUUCGGAAGACCUCGGCCCC	734

[a] The percentage representation in the RISC-associated small RNA library is shown for the most abundant MM-H read.
