# Supplementary material for: Search for MicroRNAs Expressed by Intracellular Bacterial Pathogens in Infected Mammalian Cells
Source: PLoS One. 2014 Sep 3;9(9):e106434. doi: 10.1371/journal.pone.0106434 (PMC4153649; doi:10.1371/journal.pone.0106434)
Supplement: Table S2 — Small RNAs derived from the MM-H homology region of M. smegmatis . (PDF) [file pone.0106434.s007.pdf]

Table S2. Small RNAs derived from the MM-H homology region of *M. smegmatis*.

Strand	Position	Length	Sequence	Read count
5p	888853-888873	21	GUCGAGGUCUUUCAGAUGGAU	56
	888853-888874	22	GUCGAGGUCUUUCAGAUGGAUG	82
	888853-888875	23	GUCGAGGUCUUUCAGAUGGAUGG	32
3p	888886-888906	21	UCCAUCAUCGGAAGACCUCGA	375
	888886-888907	22	UCCAUCAUCGGAAGACCUCGAC	262
	888886-888908	23	UCCAUCAUCGGAAGACCUCGACC	704
	888886-888909	24	UCCAUCAUCGGAAGACCUCGACCC	203
	888888-888906	19	CAUCAUCGGAAGACCUCGA	423
	888888-888907	20	CAUCAUCGGAAGACCUCGAC	264
	888888-888908	21	CAUCAUCGGAAGACCUCGACC	474
	888888-888909	22	CAUCAUCGGAAGACCUCGACCC	164
	888888-888910	23	CAUCAUCGGAAGACCUCGACCCC	76
	888889-888906	18	AUCAUCGGAAGACCUCGA	322
	888889-888907	19	AUCAUCGGAAGACCUCGAC	366
	**888889-888908**	**20**	AUCAUCGGAAGACCUCGACC	**889 (0.006%)**[a]
	888889-888909	21	AUCAUCGGAAGACCUCGACCC	595
	888889-888910	22	AUCAUCGGAAGACCUCGACCCC	304
	888889-888911	23	AUCAUCGGAAGACCUCGACCCCU	98

[a] The percentage representation in the RISC-associated small RNA library is shown for the most abundant read.
